# Supplementary material for: Improving accuracy and precision of heritability estimation in twin studies through hierarchical modeling: reassessing the measurement error assumption
Source: Front Genet. 2025 Apr 2;16:1522729. doi: 10.3389/fgene.2025.1522729 (PMC11999965; doi:10.3389/fgene.2025.1522729)
Supplement: Supplementary file 1 [file Presentation1.pdf]

Supplemental Material for: Improving accuracy and precision of heritability estimation in twin studies through hierarchical modeling: Reassessing the measurement error assumption

Gang Chen*[a], Dustin Moraczewski[b], and Paul A. Taylor[a]

[a]Scientific and Statistical Computing Core, National Institute of Mental Health, USA
[b]Data Science and Sharing Team, National Institute of Mental Health, USA

Simulation specifications

Simulations were conducted with the following specifications under the HLM formulation (formulation 22) compared to the conventional SEM (formulation 2). All distributions involved were assumed to be Gaussian, and the data were aggregated across trials for each condition when applied to the SEM formulation. While the Gaussian distribution is adopted here for simulation convenience and may not always be applicable in real-world scenarios, the simulation results still provide valuable insights into the impact of intra-individual variability and the necessary sample size requirements. The two overall effects under the HLM (formulation 20) were fixed at $\alpha_c = 0$ $(c = c_1, c_2)$. Since the standard deviation is essentially a scaling factor, the sum of inter-individual and inter-family variances was fixed as $\sigma_{cz}^2 + \tau_{cz}^2 = 1$ $(c = c_1, c_2$; $z =$ MZ, DZ) without loss of generality. Four factors were changed to create the set of simulations across the following ranges of values:

(a) Three family sample sizes $F = 100, 500, 1000$, half of which were MZ (and DZ) zygosities.
(b) Three trial sample sizes $T = 50, 100, 500$.
(c) Three ratios of $R_v = 1, 4, 10$, leading to $\sigma_{cz}^2 = R_v$.
(d) Two modeling approaches: SEM and HLM.

Each of these $3 \times 3 \times 3 \times 2 = 54$ combinations was simulated with 200 repetitions. For SEM, the function `lme` in the `R` package `nlme` (Pinheiro et al., 2022) was used to estimate variances. For HLM, the function `lmer` in the `R` package `lme4` (Bates et al., 2015) was adopted with the following iterative steps. The condition contrast was parameterized through the indicator variable defined in Subsection 3.5.

1) For each twin pair (i_1, i_2) within family f of zygosity z, obtain their effects at the two conditions $c1$ and c_2 through randomly sampling from the following quadrivariate distribution:

$$\begin{bmatrix} \theta_{c_1 i_1 (f(z))} \\ \theta_{c_1 i_2 (f(z))} \\ \theta_{c_2 i_1 (f(z))} \\ \theta_{c_2 i_2 (f(z))} \end{bmatrix} \sim \mathscr{N} \left(\begin{bmatrix} 0 \\ 0 \\ 0 \\ 0 \end{bmatrix}, \begin{bmatrix} 1 & \gamma_1 & \gamma_2 & \rho_z \\ \gamma_1 & 1 & \rho_z & \gamma_2 \\ \gamma_2 & \rho_z & 1 & \gamma_1 \\ \rho_z & \gamma_2 & \gamma_1 & 1 \end{bmatrix} \right).$$

Here, γ_1 and γ_2 were fixed at 0.8 and 0.5, respectively, and ρ_z was chosen as 0.375 when $z =$ MZ and 0.5 when $z =$ DZ, so that the simulated correlation rz between a twin pair within a family regarding the condition contrast would be 0.85 for $z =$ MZ and 0.6 for $z =$ DZ, respectively. Thus, the targeted heritability h^2 would be 0.5 per Falconer's formula 6, while c^2 and e^2 would be 0.35 and 0.15.

2) Randomly draw trial-level data $y_{ci(f(z))}$ from $\mathscr{N}(\alpha_c + \theta_{ci(f(z))},\ \sigma_0^2)$ $(c = c_1, c_2)$, where $\sigma_0^2 = R_v^2$.

*Corresponding author. E-mail address: gangchen@mail.nih.gov

3) Estimate σ_z^2 and τ_z^2. For SEM, obtain the average across trials for each condition and for each individual, and then apply the individual-level contrast between the conditions to the formulation 7 using the function `lme` from the `R` package `nlme`. For HLM, apply the model formulation 20 using the function `lmer` from the `R` package `lme4`.
4) Obtain r_z using the formula 21. In addition, make two adjustments using formulas 17 and 18 with $1/T$ replaced by $2/T$.
5) Estimate h^2, c^2, and e^2 using Falconer's formula 6.
6) Estimate bias adjustments for SEM: apply formula 18 with an extra factor of 2 to estimate SEM1, and formula 17 with an extra factor of 2 to estimate SEM2.

The results are shown in Figs. 1 and 2 in the Supplemental Material. The simulation scripts can be found online at https://github.com/afni/apaper_heritability.

References

Bates, D., Mächler, M., Bolker, B., Walker, S., 2015. Fitting Linear Mixed-Effects Models Using lme4. Journal of Statistical Software 67, 1–48.

Pinheiro, J., Bates, D., R Core Team, 2022. nlme: Linear and Nonlinear Mixed Effects Models. URL: `https://CRAN.R-project.org/package=nlme`. r package version 3.1-159.

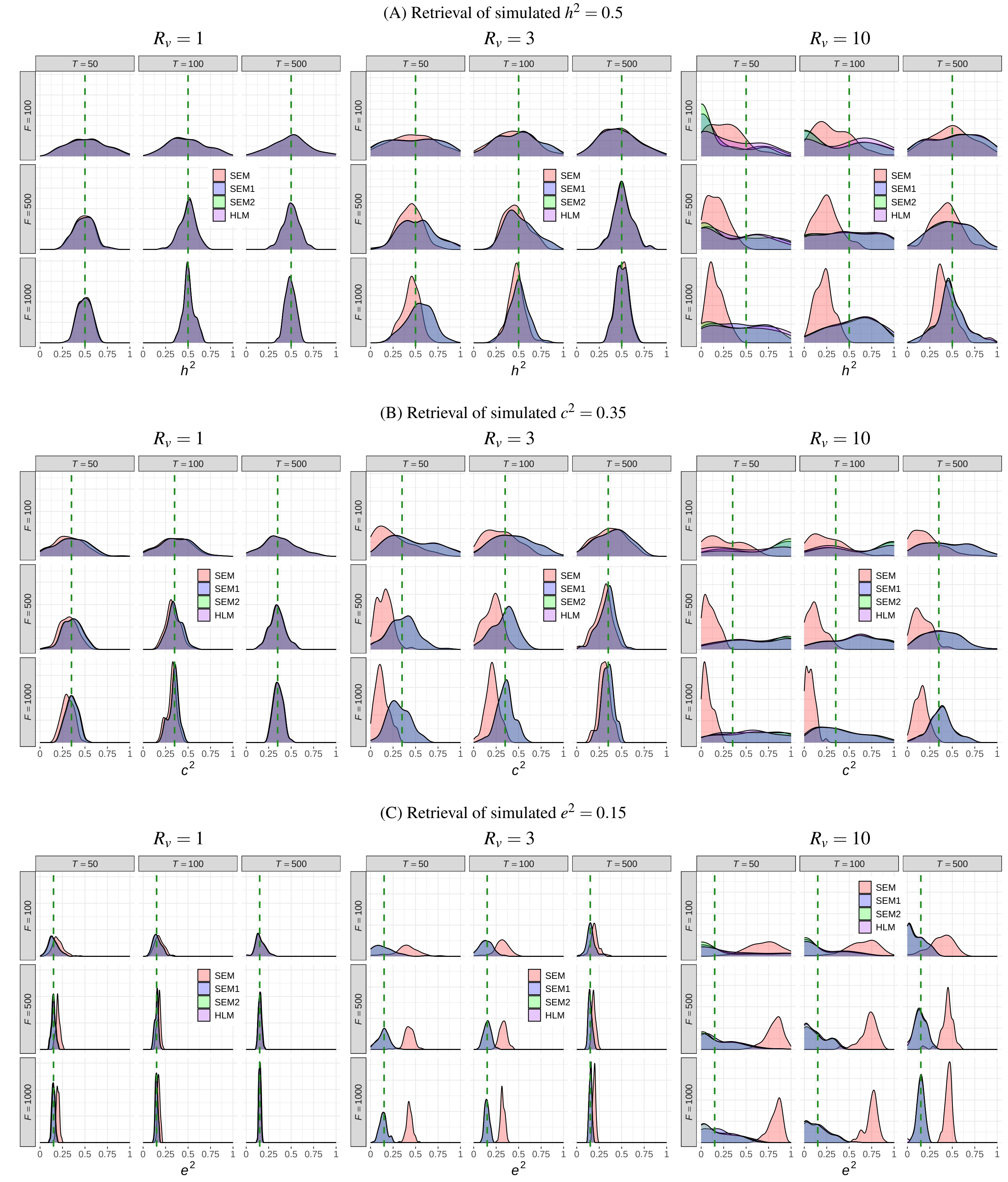

Figure 1: Simulation results of a trial-based experiment for the contrast between two conditions. Four factors were considered in the simulations: two models (SEM and HLM), three variability ratios ($R_v = 1, 4, 10$), three family sizes ($F = 100, 500, 1000$), and three trial sample sizes ($T = 50, 100, 500$). SEM1 and SEM2 represent the adjusted estimates obtained through the decontamination formulas 18 and 17, respectively. Each curve represents the density of parameter estimation from 200 repetitions, and each vertical dashed line indicates the simulated parameter value. The curves for HLM, SEM1, and SEM2 are nearly indistinguishable from each other.

(A) Retrieval of simulated $h^2 = 0.5$

F	model	$R_v = 1$			$R_v = 3$			$R_v = 10$		
		$T = 50$	$T = 100$	$T = 500$	$T = 50$	$T = 100$	$T = 500$	$T = 50$	$T = 100$	$T = 500$
100	SEM	0.57 (0.16, 0.93)	0.39 (0.1, 0.92)	0.53 (0.01, 0.99)	0.48 (0.00, 1.00)	0.44 (0.00, 1.00)	0.48 (0.06, 0.87)	0.08 (0.00, 0.56)	0.20 (0.00, 0.77)	0.50 (0.00, 1.00)
	SEM1	0.55 (0.13, 0.94)	0.38 (0.09, 0.91)	0.53 (0.01, 0.99)	0.58 (0.00, 1.00)	0.54 (0.00, 1.00)	0.43 (0.07, 0.91)	0.02 (0.00, 1.00)	0.03 (0.00, 1.00)	0.64 (0.00, 1.00)
	SEM2	0.56 (0.13, 0.94)	0.39 (0.09, 0.91)	0.53 (0.01, 0.99)	0.59 (0.00, 1.00)	0.53 (0.00, 1.00)	0.43 (0.07, 0.9)	0.01 (0.00, 1.00)	0.02 (0.00, 1.00)	0.64 (0.00, 1.00)
	HLM	0.56 (0.15, 0.97)	0.39 (0.09, 0.91)	0.53 (0.01, 0.99)	0.60 (0.00, 1.00)	0.54 (0.00, 1.00)	0.44 (0.07, 0.94)	0.03 (0.00, 1.00)	0.07 (0.00, 1.00)	0.67 (0.00, 1.00)
500	SEM	0.49 (0.26, 0.64)	0.51 (0.3, 0.64)	0.49 (0.36, 0.72)	0.46 (0.18, 0.64)	0.45 (0.27, 0.73)	0.50 (0.31, 0.71)	0.05 (0.00, 0.39)	0.24 (0.00, 0.5)	0.45 (0.18, 0.83)
	SEM1	0.53 (0.28, 0.65)	0.52 (0.33, 0.68)	0.49 (0.36, 0.72)	0.58 (0.23, 0.94)	0.42 (0.28, 0.89)	0.50 (0.31, 0.71)	0.00 (0.00, 1.00)	0.48 (0.00, 1.00)	0.45 (0.04, 1.00)
	SEM2	0.53 (0.28, 0.65)	0.52 (0.33, 0.68)	0.49 (0.36, 0.72)	0.58 (0.23, 0.94)	0.42 (0.28, 0.88)	0.50 (0.31, 0.71)	0.00 (0.00, 1.00)	0.14 (0.00, 1.00)	0.45 (0.04, 1.00)
	HLM	0.53 (0.28, 0.65)	0.52 (0.33, 0.68)	0.49 (0.36, 0.72)	0.58 (0.23, 0.96)	0.42 (0.28, 0.88)	0.50 (0.31, 0.71)	0.00 (0.00, 1.00)	0.22 (0.00, 1.00)	0.48 (0.00, 1.00)
1000	SEM	0.52 (0.36, 0.61)	0.49 (0.41, 0.65)	0.48 (0.4, 0.6)	0.46 (0.26, 0.61)	0.48 (0.29, 0.63)	0.53 (0.35, 0.63)	0.11 (0.00, 0.34)	0.24 (0, 0.4)	0.36 (0.19, 0.62)
	SEM1	0.50 (0.36, 0.62)	0.50 (0.41, 0.66)	0.48 (0.4, 0.6)	0.54 (0.25, 0.81)	0.50 (0.28, 0.7)	0.47 (0.36, 0.63)	0.13 (0.00, 1.00)	0.68 (0.00, 1.00)	0.46 (0.16, 0.81)
	SEM2	0.50 (0.36, 0.62)	0.50 (0.41, 0.66)	0.48 (0.4, 0.6)	0.54 (0.25, 0.81)	0.50 (0.28, 0.7)	0.47 (0.36, 0.63)	0.04 (0.00, 1.00)	0.67 (0.00, 1.00)	0.46 (0.16, 0.81)
	HLM	0.50 (0.36, 0.62)	0.49 (0.41, 0.66)	0.48 (0.4, 0.6)	0.54 (0.25, 0.81)	0.50 (0.28, 0.7)	0.47 (0.36, 0.63)	0.02 (0.00, 1.00)	0.67 (0.00, 1.00)	0.45 (0.16, 0.93)

(B) Retrieval of simulated $c^2 = 0.35$

F	model	$R_v = 1$			$R_v = 3$			$R_v = 10$		
		$T = 50$	$T = 100$	$T = 500$	$T = 50$	$T = 100$	$T = 500$	$T = 50$	$T = 100$	$T = 500$
100	SEM	0.28 (0.00, 0.60)	0.32 (0.00, 0.7)	0.31 (0.00, 0.81)	0.10 (0.00, 0.65)	0.19 (0.00, 0.66)	0.39 (0.00, 0.66)	0.00 (0.00, 0.59)	0.00 (0.00, 0.64)	0.10 (0.00, 0.61)
	SEM1	0.34 (0.00, 0.66)	0.32 (0.00, 0.74)	0.31 (0.00, 0.82)	0.29 (0.00, 1.00)	0.38 (0.00, 0.87)	0.45 (0.00, 0.74)	1.00 (0.00, 1.00)	1.00 (0.00, 1.00)	0.29 (0.00, 1.00)
	SEM2	0.33 (0.00, 0.66)	0.32 (0.00, 0.73)	0.31 (0.00, 0.82)	0.29 (0.00, 1.00)	0.38 (0.00, 0.87)	0.45 (0.00, 0.74)	1.00 (0.00, 1.00)	1.00 (0.00, 1.00)	0.30 (0.00, 1.00)
	HLM	0.33 (0.00, 0.69)	0.32 (0.00, 0.73)	0.31 (0.00, 0.82)	0.30 (0.00, 1.00)	0.37 (0.00, 0.87)	0.45 (0.00, 0.73)	0.98 (0.00, 1.00)	1.00 (0.00, 1.00)	0.27 (0.00, 1.00)
500	SEM	0.33 (0.15, 0.51)	0.31 (0.18, 0.46)	0.34 (0.21, 0.55)	0.17 (0.00, 0.30)	0.24 (0.00, 0.41)	0.33 (0.12, 0.46)	0.01 (0.00, 0.25)	0.00 (0.00, 0.3)	0.10 (0.00, 0.36)
	SEM1	0.38 (0.19, 0.57)	0.33 (0.21, 0.5)	0.34 (0.22, 0.56)	0.42 (0.00, 0.64)	0.39 (0.02, 0.57)	0.37 (0.15, 0.5)	0.95 (0.00, 1.00)	0.64 (0.00, 1.00)	0.37 (0.00, 0.78)
	SEM2	0.37 (0.19, 0.56)	0.33 (0.2, 0.5)	0.34 (0.22, 0.56)	0.42 (0.00, 0.64)	0.39 (0.02, 0.57)	0.37 (0.15, 0.5)	1.00 (0.00, 1.00)	0.70 (0.00, 1.00)	0.37 (0.00, 0.78)
	HLM	0.37 (0.19, 0.56)	0.33 (0.2, 0.5)	0.34 (0.22, 0.56)	0.42 (0.00, 0.64)	0.39 (0.02, 0.57)	0.37 (0.15, 0.5)	1.00 (0.00, 1.00)	0.70 (0.00, 1.00)	0.35 (0.00, 0.82)
1000	SEM	0.29 (0.17, 0.41)	0.33 (0.2, 0.41)	0.34 (0.25, 0.44)	0.10 (0.00, 0.30)	0.20 (0.08, 0.39)	0.31 (0.17, 0.44)	0.01 (0.00, 0.18)	0.00 (0.00, 0.16)	0.17 (0.00, 0.3)
	SEM1	0.35 (0.23, 0.48)	0.36 (0.23, 0.45)	0.35 (0.26, 0.45)	0.26 (0.12, 0.61)	0.37 (0.15, 0.56)	0.36 (0.2, 0.48)	0.68 (0.00, 1.00)	0.17 (0.00, 1.00)	0.39 (0.11, 0.73)
	SEM2	0.35 (0.23, 0.48)	0.35 (0.23, 0.44)	0.34 (0.26, 0.44)	0.26 (0.12, 0.61)	0.36 (0.15, 0.55)	0.36 (0.2, 0.48)	0.93 (0.00, 1.00)	0.18 (0.00, 1.00)	0.39 (0.11, 0.73)
	HLM	0.35 (0.23, 0.48)	0.35 (0.26, 0.44)	0.34 (0.23, 0.44)	0.26 (0.12, 0.61)	0.36 (0.15, 0.55)	0.36 (0.2, 0.48)	0.95 (0.00, 1.00)	0.19 (0.00, 1.00)	0.39 (0.03, 0.73)

(C) Retrieval of simulated $e^2 = 0.15$

F	model	$R_v = 1$			$R_v = 3$			$R_v = 10$		
		$T = 50$	$T = 100$	$T = 500$	$T = 50$	$T = 100$	$T = 500$	$T = 50$	$T = 100$	$T = 500$
100	SEM	0.18 (0.08, 0.34)	0.17 (0.08, 0.28)	0.14 (0.02, 0.31)	0.4 (0.00, 0.63)	0.32 (0.01, 0.5)	0.19 (0.09, 0.33)	0.76 (0.39, 1)	0.75 (0.23, 0.94)	0.44 (0.07, 0.63)
	SEM1	0.12 (0.02, 0.28)	0.14 (0.06, 0.25)	0.13 (0.01, 0.3)	0.08 (0.00, 0.4)	0.13 (0.00, 0.34)	0.15 (0.05, 0.28)	0.00 (0.00, 1.00)	0.00 (0.00, 0.59)	0.02 0.00, 0.32
	SEM2	0.13 (0.03, 0.28)	0.14 (0.07, 0.25)	0.13 (0.01, 0.3)	0.08 (0.00, 0.4)	0.14 (0.00, 0.34)	0.15 (0.06, 0.28)	0.00 (0.00, 1.00)	0.00 (0.00, 0.57)	0.02 (0.00, 0.32
	HLM	0.13 (0.03, 0.28)	0.14 (0.07, 0.25)	0.13 (0.01, 0.3)	0.08 (0.00, 0.39)	0.13 (0.00, 0.34)	0.15 (0.06, 0.3)	0.00 (0.00, 1.00)	0.00 (0.00, 0.58)	0.02 (0.00, 0.32
500	SEM	0.19 (0.15, 0.24)	0.18 (0.13, 0.2)	0.16 (0.13, 0.19)	0.42 (0.35, 0.57)	0.34 (0.24, 0.41)	0.19 (0.16, 0.23)	0.86 (0.63, 0.97)	0.74 (0.5, 0.88)	0.45 (0.28, 0.57)
	SEM1	0.14 (0.1, 0.18)	0.15 (0.11, 0.18)	0.15 (0.12, 0.19)	0.15 (0.01, 0.25)	0.16 (0.07, 0.23)	0.14 (0.11, 0.19)	0.02 (0.00, 0.57)	0.06 (0.00, 0.38)	0.13 (0.00, 0.31)
	SEM2	0.15 (0.11, 0.19)	0.16 (0.11, 0.19)	0.15 (0.12, 0.19)	0.15 (0.01, 0.25)	0.16 (0.07, 0.23)	0.14 (0.11, 0.19)	0.00 (0.00, 0.5)	0.05 (0.00, 0.37)	0.13 (0.00, 0.31)
	HLM	0.15 (0.11, 0.19)	0.16 (0.11, 0.19)	0.15 (0.12, 0.19)	0.15 (0.01, 0.25)	0.16 (0.07, 0.23)	0.14 (0.11, 0.19)	0.00 (0.00, 0.49)	0.04 (0.80, 0.37)	0.13 (0.00, 0.31)
1000	SEM	0.19 (0.16, 0.23)	0.17 (0.15, 0.21)	0.16 (0.14, 0.18)	0.42 (0.38, 0.5)	0.31 (0.28, 0.37)	0.2 (0.17, 0.22)	0.87 (0.67, 0.93)	0.77 (0.6, 0.86)	0.47 (0.37, 0.51)
	SEM1	0.14 (0.12, 0.18)	0.15 (0.13, 0.18)	0.15 (0.13, 0.17)	0.15 (0.07, 0.23)	0.14 (0.1, 0.2)	0.16 (0.12, 0.18)	0.10 (0.00, 0.5)	0.01 (0.00, 0.56)	0.15 (0.08, 0.24)
	SEM2	0.15 (0.13, 0.19)	0.15 (0.13, 0.18)	0.15 (0.13, 0.17)	0.14 (0.07, 0.23)	0.14 (0.11, 0.2)	0.16 (0.12, 0.18)	0.02 (0.00, 0.57)	0.01 (0.00, 0.55)	0.15 (0.08, 0.24)
	HLM	0.15 (0.12, 0.19)	0.15 (0.13, 0.18)	0.15 (0.13, 0.17)	0.15 (0.07, 0.23)	0.14 (0.11, 0.2)	0.16 (0.12, 0.18)	0.02 (0.00, 0.44)	0.00 (0.00, 0.42)	0.15 (0.04, 0.24)

Figure 2: Summary of simulation results. The reported point estimates represent the modes, while the 95% uncertainty intervals, shown in parentheses, are highest density intervals derived from the simulations, as illustrated in Fig. 1 in the Supplemental Material.
